# A Chemomechanobiological Model of the Long-Term Healing Response of Arterial Tissue to a Clamping Injury

**DOI:** 10.3389/fbioe.2020.589889

**Published:** 2021-01-26

**Authors:** Lauranne Maes, Julie Vastmans, Stéphane Avril, Nele Famaey

**Affiliations:** ^1^Biomechanics Section, Department of Mechanical Engineering, KU Leuven, Leuven, Belgium; ^2^Mines Saint-Etienne, Université de Lyon, Université Jean Monnet, INSERM, Saint-Étienne, France

**Keywords:** phenotype switch, vascular remodeling, smooth muscle cells (SMC), vascular clamping, myograph, finite elements

## Abstract

Vascular clamping often causes injury to arterial tissue, leading to a cascade of cellular and extracellular events. A reliable *in silico* prediction of these processes following vascular injury could help us to increase our understanding thereof, and eventually optimize surgical techniques or drug delivery to minimize the amount of long-term damage. However, the complexity and interdependency of these events make translation into constitutive laws and their numerical implementation particularly challenging. We introduce a finite element simulation of arterial clamping taking into account acute endothelial denudation, damage to extracellular matrix, and smooth muscle cell loss. The model captures how this causes tissue inflammation and deviation from mechanical homeostasis, both triggering vascular remodeling. A number of cellular processes are modeled, aiming at restoring this homeostasis, i.e., smooth muscle cell phenotype switching, proliferation, migration, and the production of extracellular matrix. We calibrated these damage and remodeling laws by comparing our numerical results to *in vivo* experimental data of clamping and healing experiments. In these same experiments, the functional integrity of the tissue was assessed through myograph tests, which were also reproduced in the present study through a novel model for vasodilator and -constrictor dependent smooth muscle contraction. The simulation results show a good agreement with the *in vivo* experiments. The computational model was then also used to simulate healing beyond the duration of the experiments in order to exploit the benefits of computational model predictions. These results showed a significant sensitivity to model parameters related to smooth muscle cell phenotypes, highlighting the pressing need to further elucidate the biological processes of smooth muscle cell phenotypic switching in the future.

## 1. Introduction

Multiple studies indicate that arterial occlusion by almost any type of clamp systematically leads to intimal injury at the site of application. For example, endothelial denudation is a widely known effect of clamping (Slayback et al., 1976; Barone et al., 1989; Margovsky et al., 1997, 1999; Hangler et al., 2008; Vural et al., 2008; Famaey et al., 2010; Geenens et al., 2016a,b). Several studies also report damage to the extracellular matrix (ECM) in the media with flattened elastic lamellae (Barone et al., 1989; Margovsky et al., 1999; Famaey et al., 2010; Geenens et al., 2016a). Moreover, healing after arterial clamping usually implies some degree of inflammation and subsequent tissue remodeling (Geenens et al., 2016a).

The tonicity of vascular smooth muscle cells (SMC) after clamping was also studied extensively (Barone et al., 1989; Famaey et al., 2010; Geenens et al., 2016b). Experimental data on the effects of arterial clamping were collected in mice (Geenens et al., 2016a,b). In this study, descending thoracic aortas were clamped at different levels of loading. Then, the aorta was either excised immediately or excised after a fixed duration of healing. After excision, rings were cut and tested with a myograph to measure the vascular tone under vasoconstriction and vasodilatation stimulations, followed by histological analyses. An acute decline of endothelium-dependent vasodilatation was observed just after clamping, but the functional response was restored after 1 month (Geenens et al., 2016b). Arterial clamping was also followed by an inflammatory response leading to some degree of fibrosis.

The role of mechanobiology in the response to arterial clamping is not clearly understood. It is known that in many conditions, vascular remodeling is mediated by the mechanical stimuli sensed by vascular SMCs, permitting to maintain wall stresses at homeostasis (Humphrey, 2002). SMCs modulate their phenotype in response to changing local environmental cues (Epstein et al., 1994), possibly performing biosynthetic, proliferative, and contractile roles in the vessel wall (Thyberg et al., 1995). Contractile SMCs react to environmental changes on the short term by contracting and relaxing to restore a homeostatic state. On the longer term, biosynthetic vascular SMC produce, and degrade the extracellular matrix, thus enabling growth and remodeling (Owens et al., 2004).

In order to decipher the role of mechanobiology in the response to arterial clamping, *in silico* predictive models can be helpful. A number of computational models for damage through overloading of soft tissues have been developed and tested by Balzani et al. (2006, 2012); Rodríguez et al. (2006); Gasser (2011); Peña (2011, 2014); Sáez et al. (2012); Famaey et al. (2013); Forsell et al. (2013); Schmidt and Balzani (2016), and Li and Holzapfel (2019). Most of these models are based on continuum damage mechanics (Kachanov, 1986; Simo and Ju, 1987), where the amount of damaged tissue is determined by a damage parameter. These models were successfully applied to predict acute damage after arterial clamping. However, most of them focused on short term fiber damage and modeled neither the active behavior of vascular SMCs nor the healing process occurring on the longer term.

Modeling the active behavior of vascular SMCs has been a topic of extensive investigation (Murtada et al., 2017), combining continuum mechanics (Murtada et al., 2010, 2012), and the kinetics of pathways involved in the active behavior (Hai and Murphy, 1988; Schmitz and Böl, 2011; Böl et al., 2012; Kida and Adachi, 2014; Liu, 2014; Bouklas et al., 2018; Ferreira et al., 2018), including phosphorylation of myosin light chain, variations of intracellular calcium concentration and membrane depolarization (Sharifimajd and Stålhand, 2014). However, to our best knowledge, none of these models depend on the concentration of specific vasoreactive agents used in myograph testing: phenylephrine (PE), acetylcholine (ACh), and sodium nitroprusside (SNP) as nitric oxide (NO) donor.

Modeling vascular healing is also rather recent. Comellas et al. (2016) presented a computational model of tissue healing after mechanical overload, in which temporal evolutions of damage are homeostasis-driven. However, no discrimination was made between the different tissue constituents (elastin, collagen, cells) in terms of damage and mechanical behavior. This can be addressed by microstructurally-motivated growth and remodeling models based on the constrained mixture model introduced by Humphrey and Rajagopal (2002). In the constrained mixture theory, the different constituents of the tissue are constrained to move together in a mixture but all have different biologically relevant stress-free states. Tissue remodeling is governed by laws of production and degradation for each constituent based on stress states. This type of model has been used to predict different tissue adaptations such as aneurysm growth for instance by Baek et al. (2006); Alberto Figueroa et al. (2009); Watton and Hill (2009); Zeinali-Davarani and Baek (2012); Valentín et al. (2013); Cyron et al. (2016); Braeu et al. (2017); Famaey et al. (2018); Latorre and Humphrey (2018b), and Mousavi et al. (2019) or wound healing by Zuo et al. (2020). However, to the best of our knowledge, the constrained mixture theory has never been used to model healing after arterial clamping.

In the present work, we aim to computationally capture the mechanobiological effects of arterial clamping. Therefore, we introduce a chemomechanical model in a constrained mixture framework, considering inflammation, collagen deposition, SMC proliferation, SMC active response as well as SMC switch from contractile to synthetic phenotype, all depending on the mechanical and chemical environment. After introducing the details of the model, we simulate the response to arterial clamping after 1 and 2 months of healing and compare the results to experimental data.

## 2. Materials and Methods

### 2.1. Mouse Experiments

As reported by Geenens et al. (2016a), 108 wildtype mice were subjected to a surgical procedure, in which the descending thoracic aorta was isolated and clamped *in vivo* with a non-serrated, 2 mm wide clamp at either a loading level of 0.0 N (control group), 0.6 N or 1.27 N. The clamped tissue was then either immediately excised, or *in vivo* healing was allowed for 6 h, 2 weeks, or 1 month. After these four time points, histological analyses were carried out to assess the structural integrity of the tissue through CD105, CD45, Verhoeff's-Van Gieson, and osteopontin—α-SMA stainings. After the immediate excision or after 1 month, myograph tests were carried out to assess the functional integrity of the tissue. The aorta segment was mounted onto two rods in an organ bath and, upon stretching of the tissue, a stable pre-load of 20 mN was reached. Afterwards, the vasoactive substances PE, ACh, and SNP were subsequentially added to the solution to assess endothelium dependent and independent vasodilation. In total, all mice that underwent surgery were divided into eighteen groups corresponding to a particular condition, depending on the clamping force and the healing time, and on the type of assessment, i.e., histology or myograph. More details on these animal experiments are given in Geenens et al. (2016a).

### 2.2. Constitutive Model

#### 2.2.1. Passive Material Behavior

The anisotropic and nonlinear passive mechanical behavior of arterial tissue is often represented by a Gasser-Ogden-Holzapfel (Gasser et al., 2006) hyperelastic formulation. The deviatoric strain energy function is decomposed in an isotropic Neo-Hookean part, representing the elastin fibers in the tissue, and an exponential, anisotropic part, representing two collagen fiber families running in two symmetric directions. Assuming a fully incompressible material and ignoring the volumetric contribution, the strain energy function of the elastin and collagen contribution is respectively written as

(1) $$\begin{array}{l} {\hat{\Psi }}^{elas}=C_{10}({\bar{I}}_{1}^{elas}-3),\\ {\hat{\Psi }}_{i}^{coll}=\frac{k_{1}}{2k_{2}}\exp\;\left\{k_{2}{\left[(\kappa {\bar{I}}_{1}^{coll}+(1-3\kappa ){\bar{I}}_{i}^{coll})-1\right]}^{2}\right\}-1,\;i=4,6, \end{array}$$

where *C*_10_ and *k*_1_ represent the stiffness of elastin and collagen. *k*_2_ determines the exponential collagen behavior and κ quantifies the fiber dispersion. ${\bar{I}}_{1}^{elas}$ and ${\bar{I}}_{1}^{coll}$ are the first invariants or traces of the deviatoric right Cauchy-Green stretch tensors $\mathrm{tr}\left({\overset{-}{\text{C}}}^{elas}\right)=\mathrm{tr}\left(J^{-2/3}{{\text{F}}^{elas}}^{T}{\text{F}}^{elas}\right)$ and $\mathrm{tr}\left({\overset{-}{\text{C}}}^{coll}\right)=\mathrm{tr}\left(J^{-2/3}{{\text{F}}^{coll}}^{T}{\text{F}}^{coll}\right)$, where ***F***^*elas*^ and ***F***^*coll*^ are the deformation gradients of elastin and collagen respectively and *J* is the Jacobian of the deformation gradient ***F***. More information on these different deformation gradients follows in section 2.2.4. ${\bar{I}}_{4}^{coll}$ and ${\bar{I}}_{6}^{coll}$ are the fourth and sixth invariants of ${\overset{-}{\text{C}}}^{coll}$ and ***M***_*i*_, representing the stretch along the preferred fiber direction, written as

(2) $$\begin{array}{l} {\bar{I}}_{i}^{coll}={\text{M}}_{i}\cdot \left({\overset{-}{C}}^{coll}{\text{M}}_{i}\right),\;i=4,6, \end{array}$$

with ***M***_*i*_ the undeformed fiber vector defined by the fiber angle α_*i*_ with respect to the circumferential direction. Therefore, ${\text{M}}_{i}={\left[0\;\cos\alpha_{i}\;\sin\alpha_{i}\right]}^{T}$, assuming that the radial direction is the first direction, the circumferential direction the second and the axial the third.

#### 2.2.2. Active Material Behavior

Contractile SMCs in the media actively generate vascular tone. An active component to the strain energy function, as described by Murtada et al. (2010) and used by Famaey et al. (2013) takes the form

(3) $$\begin{array}{l} {\hat{\text{Ψ}}}^{csmc}=\frac{\mu_{smc}}{2}\left(n_{3}+n_{4}\right){\left(\sqrt{{\bar{I}}_{4}^{smc}}+u_{rs}-1\right)}^{2}, \end{array}$$

where μ_*smc*_ is a stiffness-like material parameter, *n*_3_ and *n*_4_ together are the fractions of the smooth muscle filaments in the force-producing states. *u*_*rs*_ represents the normalized sliding between the filaments arising from the difference between the stress in the surrounding matrix *P*_*mat*_ and the driving stresses of the cross-bridges of the filaments *P*_*smc*_. Murtada et al. (2010) give an in-depth explanation of these variables. This is also further elaborated in section 2.5. ${\bar{I}}_{4}^{smc}$ is the fourth invariant of ***M***_*smc*_ and ${\overset{-}{\text{C}}}^{smc}=J^{-2/3}{{\text{F}}^{smc}}^{T}{\text{F}}^{smc}$, the deviatoric right Cauchy-Green stretch tensor of the smooth muscle fibers associated with the smooth muscle deformation gradient ***F***^*smc*^. ${\bar{I}}_{4}^{smc}$ can be written similarly to Equation (2), where ***M***_*smc*_ represents the orientation of the cells. Assuming that the cells are aligned along the circumferential direction, we write ${\text{M}}_{smc}={\left[0\;1\;0\right]}^{T}$.

#### 2.2.3. Strain Energy Function

Similarly to Famaey et al. (2018), the overall strain energy density stored in the material is calculated with a mass-averaged rule as

(4) $$\begin{array}{l} \Psi =\Psi^{elas}+\Psi^{coll}+\Psi^{csmc}\\ \;=\rho^{elas}(k){\hat{\Psi }}^{elas}(k)+\sum_{\tau =0}^{k}\rho^{coll}(k,\tau ){\hat{\Psi }}_{i}^{coll}(k,\tau )\\ \;+\rho^{csmc}(k){\hat{\Psi }}^{csmc}(k), \end{array}$$

where ρ^*elas*^(*k*) represents the elastin density at the current time step *k*. ρ^*csmc*^(*k*) is the density of SMCs in their contractile phenotype and ρ^*coll*^(*k*, τ) is the density of collagen cohort τ. These considered densities and constituent specific strain energy densities relate to the reference configuration. The deposition of collagen is discretized, such that different collagen cohorts can be identified, depending on the time of production. We consider all the initially present collagen as one cohort deposited at *k* = 0. On top of that, at every discrete time step, one cohort per collagen family is produced. At every time step, all existing cohorts, for example previously deposited at time step τ, are degraded through a slowly decaying survival fraction. In the present study, we consider two symmetric fiber families, each divided into *k* + 1 cohorts at every time step *k*.

#### 2.2.4. Deformation Gradient

The strain energy defined in Equation (4) depends on the deformation gradients of the considered constituents. According to the constrained mixture growth and remodeling theory, the total deformation gradient of elastin ***F***^*elas*^ is written as

(5) $$\begin{array}{l} {\text{F}}^{elas}=\text{F}G^{elas}, \end{array}$$

where ***G***^*elas*^ is a deformation gradient containing the deposition stretches of elastin in the *in vivo* homeostatic reference state of the artery and ***F*** represents any deformation of the mixture as a whole with respect to this reference.

The total deformation gradient of a certain collagen cohort τ is

(6) $$\begin{array}{l} {\text{F}}^{coll}\left(\tau \right)=\text{F}{\text{F}}_{dep}^{coll}{\left(\tau \right)}^{-1}G^{coll}. \end{array}$$

***G***^*coll*^ represents the deformation of the collagen cohort at deposition. ${\text{F}}_{dep}^{coll}\left(\tau \right)$ is the deformation of the mixture at the time of deposition with respect to the homeostatic reference state and ***F*** is the current deformation. In a steady state regime, the deformation at deposition of all collagen still present is equal to the current deformation, such that ${\text{F}}_{dep}^{coll}=\text{F}$ and that ***F***^*coll*^ is simply equal to ***G***^*coll*^, the collagen deposition stretch tensor. Collagen is assumed to be deposited at a constant stretch *g*^*coll*^ (Bellini et al., 2014) along the main fiber direction. Therefore, for a particular fiber direction ***M*** (Cyron et al., 2016),

(7) $$\begin{array}{l} G^{coll}=g^{coll}\text{M}⊗\text{M}+\frac{1}{\sqrt{g^{coll}}}\left(\text{I}-\text{M}⊗\text{M}\right). \end{array}$$

Contractile SMCs are assumed to only feel the deformation with respect to the state at which they were deposited. Therefore, their deformation gradient is

(8) $$\begin{array}{l} {\text{F}}^{csmc}\left(\tau \right)=\text{F}{\text{F}}_{dep}^{csmc}{\left(\tau \right)}^{-1}, \end{array}$$

where ${\text{F}}_{dep}^{csmc}\left(\tau \right)$ is the deformation gradient of the mixture at the time of deposition τ of the considered cohort.

All deformations are considered fully incompressible. Moreover, volumetric changes due to mass addition or loss are neglected, such that no deformation is observed as a result of growth.

### 2.3. Damage Model

**Figure 1:**
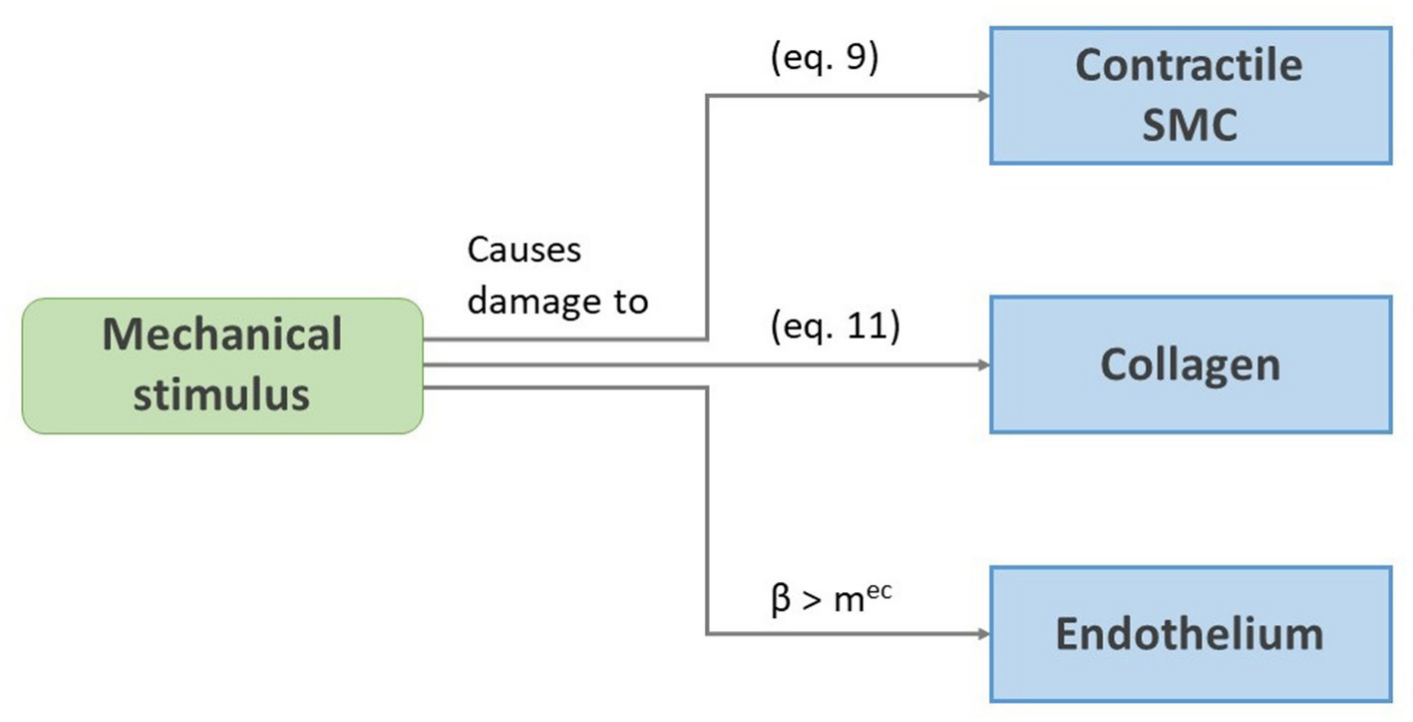
Schematic representation of the damage effects presented in section 2.3.

Figure 1 gives an overview of the considered damage effects. A short-term damage model for contractile SMCs and collagen inspired by Famaey et al. (2013) and Balzani et al. (2006) is considered. The fraction of damaged cells is modeled as a damage parameter *d*^*csmc*^, calculated as

(9) $$\begin{array}{l} d^{csmc}=1-\exp\left(-\beta /m^{csmc}\right), \end{array}$$

where *m*^*csmc*^ is a damage constant and

(10) $$\begin{array}{l} \beta =\max abs\left(\lambda_{\theta \theta }-1\right), \end{array}$$

where λ_θθ_ is the local circumferential stretch with respect to the *in vivo* reference stretch, assuming that the deformation gradient is known in a predefined local coordinate system whose axes are aligned with the local radial, circumferential, and axial directions.

The fraction of damaged collagen *d*^*coll*^ becomes

(11) $$\begin{array}{l} d^{coll}=1-\exp\left(-\Delta \zeta /m^{coll}\right), \end{array}$$

where again *m*^*coll*^ is a constant. Δζ is calculated as the difference between the current and homeostatic fiber stresses (see also Equation 13).

We assume that endothelium can be damaged as a result of overloading of the inner elastin of the media, since the endothelium itself bears almost no load. As stated by Jufri et al. (2015), endothelial cells react differently to physiological and pathological ranges of mechanical stretch, where the latter may induce apoptosis (Kou et al., 2009). We therefore assume that the local endothelium dies if a certain threshold *m*^*ec*^ of the local β is exceeded.

### 2.4. Remodeling Model

**Figure 2:**
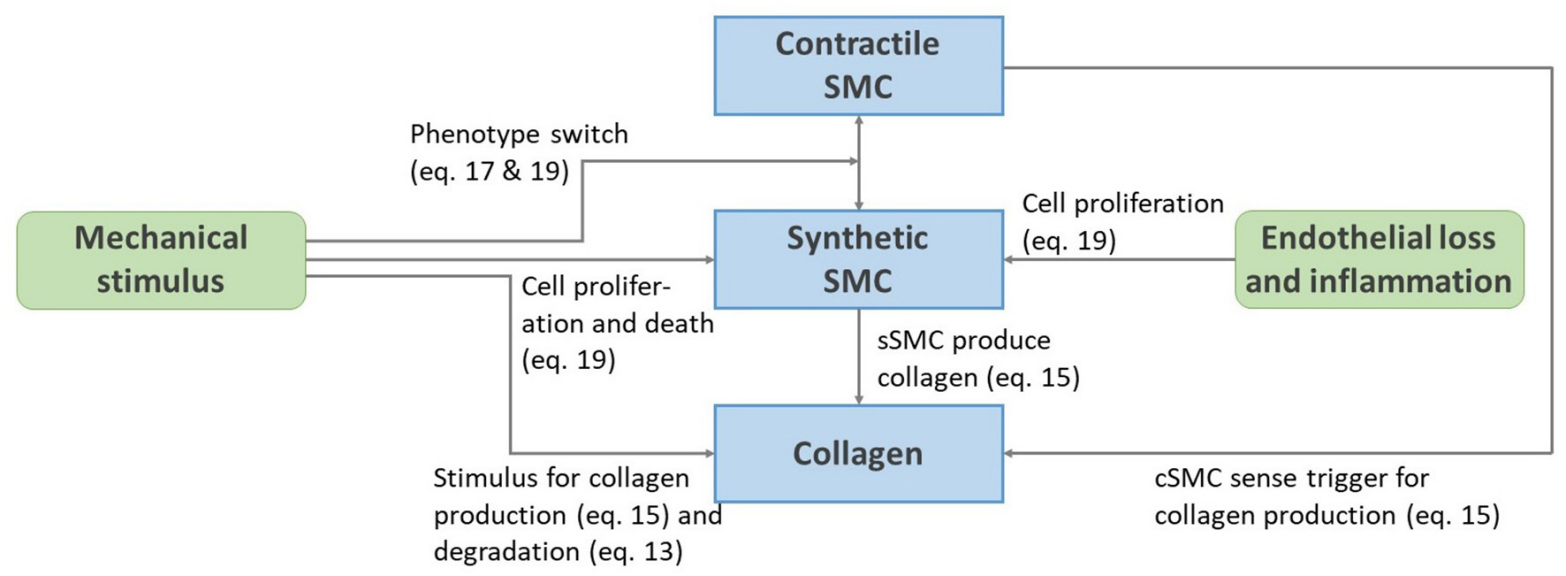
Schematic representation of the remodeling pathways presented from Equations (13) to (22).

A remodeling algorithm is defined considering six main components. In the following sections, remodeling pathways are introduced for the two main passive load-bearing constituents elastin and collagen. Two SMC phenotypes are considered, active load-bearing contractile cells and non-load-bearing synthetic cells that produce extracellular matrix. We also consider the healing of the endothelium and the infiltration of inflammatory agents. The scheme on Figure 2 is an overview of the remodeling pathways with the corresponding equations introduced in the following sections.

#### 2.4.1. Elastin

We assume that the production or degradation of elastin is negligible over the considered time frame and that new elastin cannot be produced. The elastin density at each time point is therefore equal to the homeostatic density $\rho_{0}^{elas}$.

#### 2.4.2. Collagen

The density at time step *k* of a collagen cohort deposited at time τ is (Valentín et al., 2013; Famaey et al., 2018)

(12) $$\begin{array}{l} \rho^{coll}\left(k,\tau \right)=m^{coll}\left(\tau \right)q^{coll}\left(k,\tau \right). \end{array}$$

*m*^*coll*^(τ) represents the amount of collagen of the specified cohort at the time of deposition τ and *q*^*coll*^(*k*, τ) is the fraction of this cohort that survives until time *k*.

The degradation of collagen depends on the current fiber stress. Upon discretization of Equation (8) in Famaey et al. (2018) or (53) in Valentín et al. (2013), the survival fraction of a certain collagen cohort is

(13) $$q^{coll}(k,\tau )=\exp(-\sum_{\tilde{\tau }=\tau }^{k}k_{qh}^{coll}\Delta t(1+\Delta \zeta {\left(k,\tilde{\tau }\right)}^{2})),$$

where $K_{qh}^{coll}$ is the homeostatic decay constant and $\Delta \zeta \left(k,\tilde{\tau }\right)$ represents the difference between the current and homeostatic fiber stresses as defined in Valentín et al. (2013) and Famaey et al. (2018).

The production of new collagen cohorts is proportional to the current density of synthetic cells. The rate at which they produce collagen depends on the presence of contractile cells and on the mechanical stimulus Δλ felt by these latter cells. The production rate at time τ is written as

(14) $$\begin{array}{l} m^{coll}\left(\tau \right)=m_{0}^{coll}\Gamma \left(\tau \right), \end{array}$$

where

(15) $$\begin{array}{l} \Gamma \left(\tau \right)=\left(1+\frac{\rho^{csmc}\left(\tau \right)}{\rho_{0}^{csmc}}K_{m}^{coll}\Delta \lambda \right)\frac{\rho^{ssmc}\left(\tau \right)}{\rho_{0}^{ssmc}}. \end{array}$$

ρ^*csmc*^ and $\rho_{0}^{csmc}$ are the current and homeostatic densities of contractile cells and ρ^*ssmc*^ and $\rho_{0}^{ssmc}$ are the corresponding densities of the synthetic cells. $K_{m}^{coll}$ is a remodeling parameter and the mechanical criterion for remodeling is written as

(16) $$\begin{array}{l} \Delta \lambda =\lambda_{\theta \theta }-1. \end{array}$$

Note that Δλ is very similar to β^*csmc*^. It can however be positive or negative for circumferential stretch or compression, respectively.

#### 2.4.3. Contractile Smooth Muscle Cells

Contractile SMCs dedifferentiate into synthetic cells upon mechanical triggering, for example as observed by Wang et al. (2018) or when losing grip to the surrounding extracellular matrix. We assume that these cells react to stretch in the circumferential direction as

(17) $$\begin{array}{l} \frac{d\rho^{csmc}}{dt}=-\Delta \lambda K_{dd}^{smc}\rho^{csmc}. \end{array}$$

Through numerical integration, this becomes

(18) $$\begin{array}{l} \rho^{csmc}\left(k\right)=\rho^{csmc}\left(k-1\right)\left(1-\Delta \lambda K_{dd}^{smc}\Delta t\right), \end{array}$$

where $K_{dd}^{smc}$ is a rate parameter for cell differentiation and Δ*t* the considered time step. Regardless of this equation, the maximum relative amount of contractile cells $\rho^{csmc}/\rho_{0}^{csmc}$ is bounded by the relative amount of elastin $\rho^{elas}/\rho_{0}^{elas}$ since we assume that cells cannot be contractile if they are unable to grip the extracellular environment. The increase of contractile SMCs is also bounded by the available amount of synthetic cells to differentiate from.

#### 2.4.4. Synthetic Smooth Muscle Cells

Whereas, contractile cells are quiescent in their normal state, synthetic SMCs are more proliferative (Hao et al., 2003). We therefore assume that their density can increase through dedifferentiation or proliferation based on the mechanical environment (Mantella et al., 2015). Moreover, their proliferation also increases as a reaction to inflammation (Yang et al., 2018). We write the evolution law of these cells as

(19) $$\begin{array}{l} \frac{d\rho^{ssmc}}{dt}=\left(\Delta \lambda K_{pl}^{smc}+K_{ic}^{smc}\phi^{ic}\right)\rho^{ssmc}+\Delta \lambda K_{dd}^{smc}\rho^{csmc}. \end{array}$$

Therefore, in discretized form, the current synthetic cell density is

(20) $$\begin{array}{l} \rho^{ssmc}\left(k\right)=\rho^{ssmc}\left(k-1\right)\left(\Delta \lambda K_{pl}^{smc}\Delta t+1+K_{ic}^{smc}\Delta t\phi^{ic}\right)\\ \;+\rho^{csmc}\left(k-1\right)-\rho^{csmc}\left(k\right), \end{array}$$

where $K_{pl}^{smc}$ and $K_{ic}^{smc}$ are rate parameters related to the mechanical and inflammatory stimulus, respectively and ϕ^*ic*^ is the current fraction of inflammation.

#### 2.4.5. Endothelial Cells

After degradation, the endothelium heals following the logistic growth law

(21) $$\begin{array}{l} \frac{d\phi^{ec}}{dt}=K^{ec}\left(1-\phi^{ec}\right)\phi^{ec} \end{array}$$

or

(22) $$\begin{array}{l} \phi^{ec}\left(k\right)=\left(K^{ec}\Delta t\left(1-\phi^{ec}\left(k-1\right)\right)+1\right)\phi^{ec}\left(k-1\right), \end{array}$$

in which *K*^*ec*^ is a rate parameter and ϕ^*ec*^ is the total fraction of endothelium present. ϕ^*ec*^ = 1 means that the endothelium is fully recovered. If ϕ^*ec*^ = 0 and no endothelial cells are present at all, no recovery is possible.

#### 2.4.6. Inflammation

We model inflammation provoked when platelets and leukocytes adhere to the de-endothelialized artery and send inflammatory agents in the tissue. We therefore assume that the inflammation is directly related to the fraction of intact endothelium:

(23) $$\begin{array}{l} \phi^{ic}=1-\phi^{ec}, \end{array}$$

where ϕ^*ic*^ represents a relative level of inflammation with a maximum of 1.

### 2.5. Contractility Model

Equation (3) describes the energy generated by SMCs. As stated before, this energy depends on the muscle filament sliding and on the fraction of the filaments in their force-producing states.

Similarly to Murtada et al. (2010) and Famaey et al. (2013), the driving equation for the evolution of the relative sliding of the myofilaments *u*_*rs*_ is

(24) $$\begin{array}{l} {\dot{u}}_{rs}=\frac{1}{\eta }\left(P_{smc}-P_{mat}\right), \end{array}$$

with

(25) $$\begin{array}{l} P_{mat}=\frac{\partial {\text{Ψ}}^{csmc}}{\partial \lambda_{\theta }}=J^{-2/3}\mu^{csmc}\left(n_{3}+n_{4}\right)\left(\sqrt{{\bar{I}}_{4}^{csmc}}+u_{rs}-1\right), \end{array}$$

and

(26) $$P_{smc}=\{\begin{array}{ll} \kappa_{c}n_{3}, & \text{for }P_{\text{mat}}<\kappa_{\text{c}}n_{3}\\ P_{mat}, & \text{for }\kappa_{\text{c}}\left(n_{3}{\text{+n}}_{\text{4}}\right)\geq P_{\text{mat}}\geq \kappa_{\text{c}}n_{3}\\ \kappa_{c}\left(n_{3}+n_{4}\right), & \text{for }P_{\text{mat}}>\kappa_{\text{c}}\left(n_{3}{\text{+n}}_{\text{4}}\right) \end{array}$$

In a steady-state or homeostatic condition, *u*_*rs*_ is evolved to a situation where *P*_*smc*_ = *P*_*mat*_. Mathematically, three situations can be discerned. Potentially, the *u*_*rs*_ value from a previous state already allows κ_*c*_ (*n*_3_ + *n*_4_) ≥ *P*_*mat*_ ≥ κ_*c*_*n*_3_ to be true in the new steady-state, such that *P*_*smc*_ = *P*_*mat*_ already holds, as can be seen from Equation (26). *u*_*rs*_ then does not evolve further in the new steady-state. The previous *u*_*rs*_ may also cause *P*_*mat*_ to be smaller than κ_*c*_*n*_3_. In that case, *u*_*rs*_ evolves until *P*_*mat*_ = κ_*c*_*n*_3_. At the final steady-state, *u*_*rs*_ is then written as

(27) $$\begin{array}{l} u_{rs}=\frac{\kappa_{c}n_{3}}{\mu_{smc}\left(n_{3}+n_{4}\right)}+1-\sqrt{{\bar{I}}_{4}^{csmc}}. \end{array}$$

Alternatively, if the previous *u*_*rs*_ causes *P*_*mat*_ to be greater than κ_*c*_(*n*_3_ + *n*_4_), *u*_*rs*_ evolves to

(28) $$\begin{array}{l} u_{rs}=\frac{\kappa_{c}}{\mu_{smc}}+1-\sqrt{{\bar{I}}_{4}^{csmc}}. \end{array}$$

The steady-state configuration of the muscle filaments can therefore be calculated at any level of deformation, quantified by Ī_4_.

As defined by Hai and Murphy (1988) and Murtada et al. (2010), the myofilaments switch between their states *n*_1_, *n*_2_, *n*_3_, or *n*_4_ by the set of differential equations

(29) $$\begin{array}{l} \left[\begin{array}{c} {ṅ}_{1}\\ {ṅ}_{2}\\ {ṅ}_{3}\\ {ṅ}_{4} \end{array}\right]=\left[\begin{array}{cccc} -k_{1} & k_{2} & 0 & k_{7}\\ k_{1} & -\left(k_{2}+k_{3}\right) & k_{4} & 0\\ 0 & k_{3} & -\left(k_{4}+k_{5}\right) & k_{6}\\ 0 & 0 & k_{5} & -\left(k_{6}+k_{7}\right) \end{array}\right]\left[\begin{array}{c} n_{1}\\ n_{2}\\ n_{3}\\ n_{4} \end{array}\right], \end{array}$$

where *n*_1_ and *n*_2_ represent the fractions of myofilaments in their detached state, while *n*_4_ and *n*_3_ represent the fractions of attached filaments, dephosphorylated, and phosphorylated, respectively. As explained by Murtada et al. (2010), the rate parameters *k*_1_ and *k*_6_ are dependent on the calcium concentration [*Ca*^2+^] using Michaelis-Menten kinetics as

(30) $$\begin{array}{l} k_{1}=k_{6}=\frac{{\left[CaCaM\right]}^{2}}{{\left[CaCaM\right]}^{2}+K_{CaCaM}^{2}}s^{-1},\left[CaCaM\right]=\alpha_{Ca}\left[Ca^{2+}\right]. \end{array}$$

The second equation represents the formation of Calcium-Calmodulin complex, where α_*Ca*_ is a positive constant. *K*_*CaCaM*_ in the first equation, inspired by Yang et al. (2003) is a CaCaM-dependent phosphorylation rate parameter.

We assume that [*Ca*^2+^] represents the intracellular calcium contraction. This concentration can be influenced by vasoactive agents, such as the vasodilator NO and the vasoconstrictor PE. The response to these agents is normalized with respect to the maximal possible response by the Hill equation:

(31) $$\begin{array}{l} R_{NO}=\frac{\left[NO\right]}{\left[NO\right]+K_{NO}} \end{array}$$

and

(32) $$\begin{array}{l} R_{PE}=\frac{\left[PE\right]}{\left[PE\right]+K_{PE}}, \end{array}$$

where [*NO*] and [*PE*] are the respective extracellular concentrations of NO and PE. *K*_*NO*_ and *K*_*PE*_ determine the inflection points of the Hill curves. PE causes an increase of the calcium concentration, whereas NO decreases it. The current intracellular calcium concentration is determined as

(33) $$\begin{array}{l} \left[Ca^{2+}\right]={\left[Ca^{2+}\right]}_{hom}+\alpha_{PE}R_{PE}-\alpha_{NO}R_{NO}, \end{array}$$

where ${\left[Ca^{2+}\right]}_{hom}$ is the homeostatic intracellular calcium concentration and α_*PE*_ is the maximum extra calcium concentration in response to PE. α_*NO*_ is the maximal calcium concentration that is removed in response to NO. Plugging the resulting [*Ca*^2+^] back into Equation (30), a NO and PE dependency of the rate parameters *k*_1_ and *k*_6_ is observed.

*k*_2_ and *k*_5_, that define the rate of dephosphorylation, are also directly affected by the NO concentration since NO activates myosin light chain phosphatase (Carvajal et al., 2000). We write

(34) $$\begin{array}{l} k_{2}=k_{5}=k_{2,hom}+\alpha_{2}R_{NO}, \end{array}$$

where *k*_2,*hom*_ is the homeostatic rate of dephosphorylation and α_2_ is the maximal increase in response to NO.

NO is produced by a healthy endothelium in response to, for example, the vasodilating agent acetylcholine (ACh) or wall shear stress (WSS). Cohen et al. (1997) measured the NO concentration in response to ACh. Their findings are approximated with the equation

(35) $$\begin{array}{l} {\left[NO\right]}_{ec}=2.8\cdot 10^{-7}\cdot \frac{\log_{10}\left(\left[ACh\right]/1M\right)+8.2}{0.9+\log_{10}\left(\left[ACh\right]/1M\right)+8.2}\cdot 1M, \end{array}$$

where [*NO*]_*ec*_ is the extracellular concentration of NO produced by the endothelium, [*ACh*] is the extracellular concentration of ACh and 1*M* refers to a concentration of one molar.

**Figure 3:**
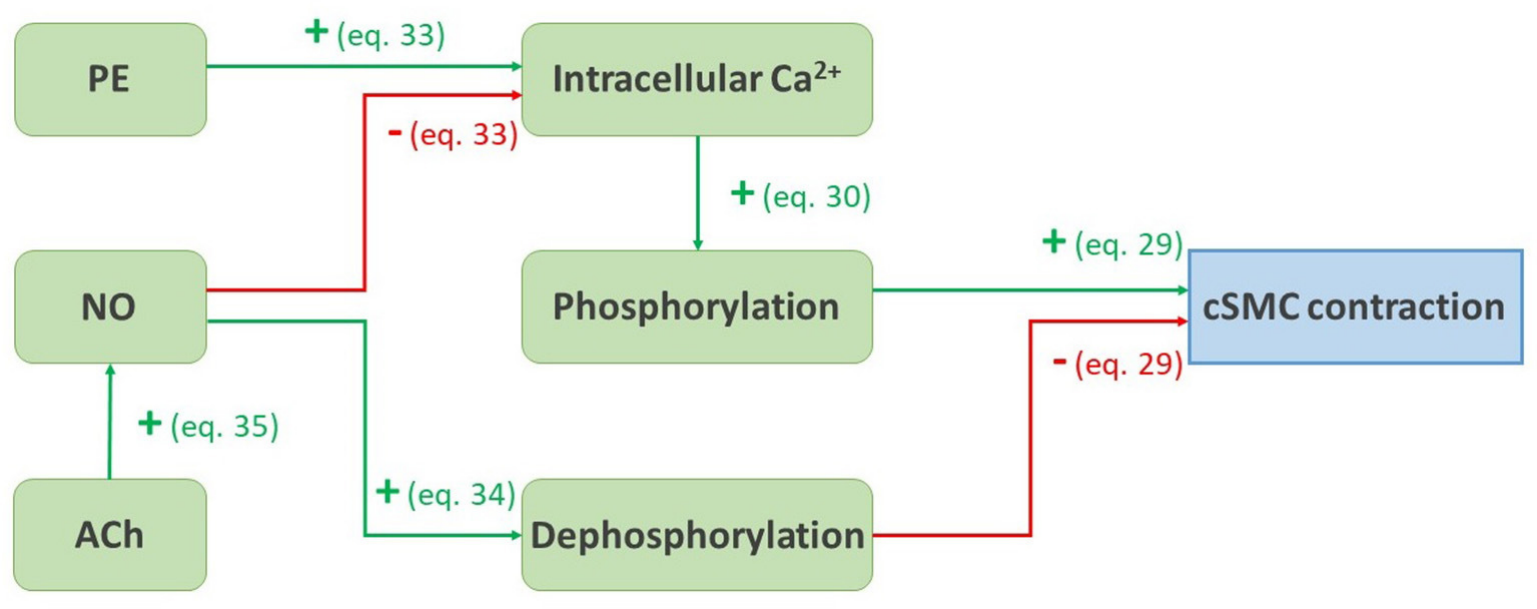
Schematic representation of the pathways presented from Equations (29) to (35). A green arrow with a plus sign represents a positive influence, a red arrow with a minus sign represents a negative influence.

In summary, the whole dependency of the contractile state of the contractile cells on the vasoactive agent PE, NO, and ACh is schematically represented in Figure 3.

### 2.6. Finite Element Model

**Figure 4:**
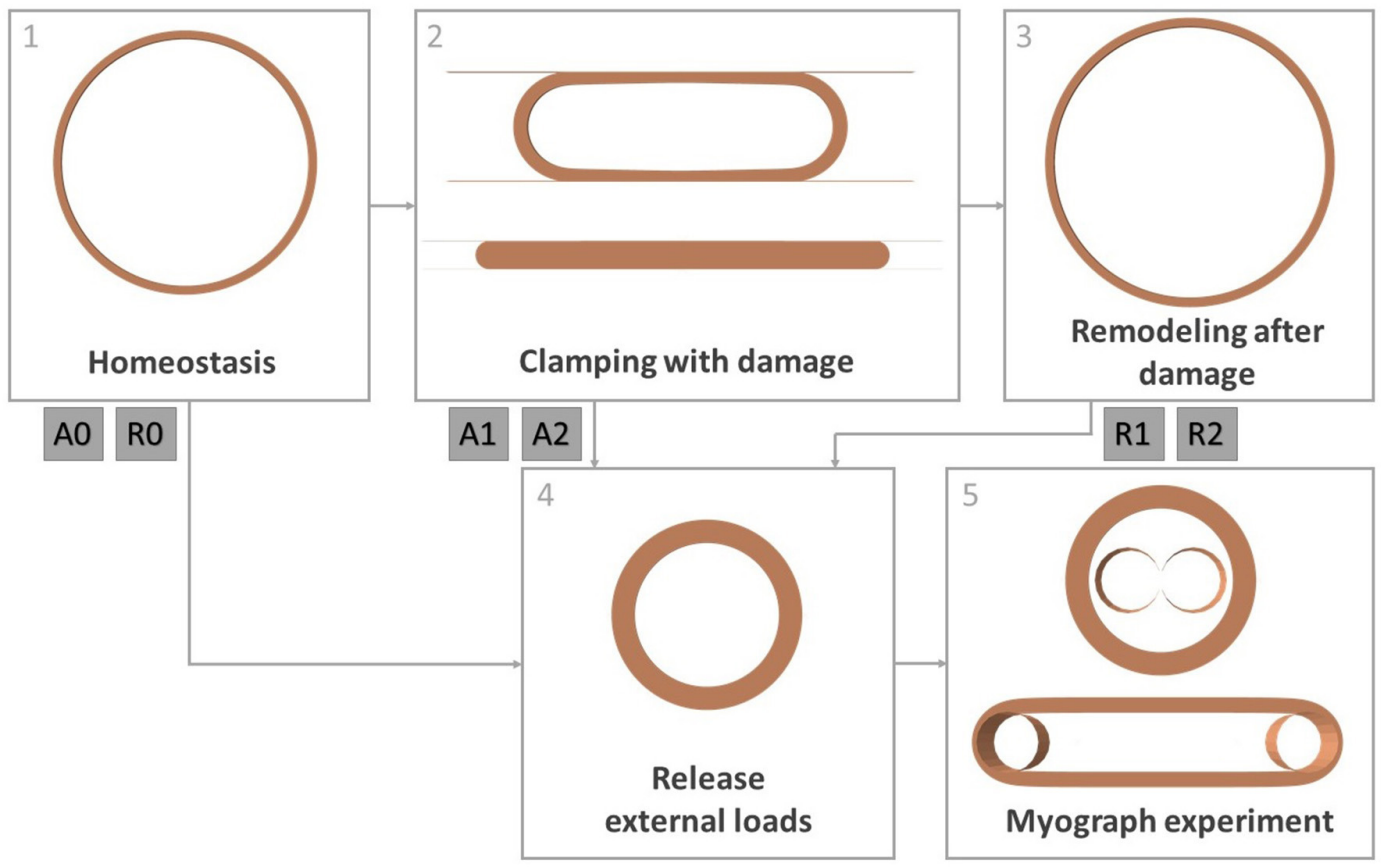
Overview of all simulation steps. Step 1: obtaining homeostatic configuration, step 2: clamping the artery while allowing damage to the constituents, step 3: remodeling of the tissue after damage, step 4: releasing intraluminal pressure and axial tension, step 5: simulation of a myograph experiment. Steps 4 and 5 are done after step 1 (cases A0 and R0), after step 2 (cases A1 and A2) and after step 3 (cases R1 and R2).

The material, damage and remodeling models explained above are used for an *in silico* reproduction of the experiments carried out by Geenens et al. (2016a). A finite element model is set up in Abaqus/Standard 2017 to represent a mouse aorta. The diastolic geometry of the aorta is represented as a half cylinder with inner diameter 0.65 mm and thickness 0.04 mm (Bersi et al., 2016). Due to symmetry, only a length of 0.04 mm of the cylinder is modeled. The geometry consists of 12,852 full integration, hexahedral, hybrid elements (C3D8H). The simulation goes through a number of steps explained below, according to the steps followed in the actual experiments. An overview is also shown in Figure 4.

#### 2.6.1. Homeostasis

In order to model the *in vivo*, mechanobiologically homeostatic condition of the mouse aorta, the prestressing algorithm explained in Mousavi and Avril (2017), Famaey et al. (2018), and Maes et al. (2019) is used. This algorithm looks for a suitable deposition stretch deformation gradient for elastin ***G***^*elas*^ in order to balance the diastolic *in vivo* reference geometry with the intraluminal diastolic pressure *p* = 10 kPa, while the top and bottom of the arterial section are fixed in axial direction. The collagen deposition stretch *g*^*coll*^ and the axial elastin deposition stretch $g_{ax}^{elas}$ are fixed as prior knowledge. This simulation is shown in Figure 4 as step 1.

#### 2.6.2. Clamping

As shown in step 2 of Figure 4, the clamping of the aorta is simulated using two undeformable parallel plates, similarly to Famaey et al. (2013) and as shown Figure 4. Self-contact of the inner surface of the artery is defined, as well as contact between the plates and the outer surface of the artery. The plates first move toward each other until reaching the desired clamping force, while no damage to the material is allowed. Two different clamp forces are discerned: 0.6 or 1.27 N per 2 mm length of the clamp, respectively named load 1 and load 2. In a few next steps, the clamps are held at a constant distance and the damage model explained in section 2.3 is activated. When the damage to the endothelium and contractile SMC has stabilized, the damage is held constant again, while the clamp plates are removed. During the whole process the intraluminal pressure is fixed at mouse aortic level *p* = 10 kPa.

#### 2.6.3. Remodeling

After the releasing of the clamp, the remodeling algorithm explained in section 2.4 is activated, while keeping the pressure constant. This is shown as step 3 in Figure 4. The local collagen and SMC densities are initialized based on the previously calculated local damage. The initial value of ϕ^*ec*^ is defined as the percentage of intact endothelial layer in the entire considered segment. Therefore, this is not a locally defined variable, and the same value is attributed to every integration point.

Due to the initial loss of contractile SMC and collagen, a dilatation of the artery is observed. This non-homeostatic mechanical state drives remodeling. Every remodeling step corresponds to 1 day of remodeling. During this process, all nodes are constrained to only move in the radial direction in order to avoid excessive shearing between the layers and failure of the simulation.

#### 2.6.4. Myograph Test

After 31 days of remodeling (cases R1 and R2 for loads 1 and 2), immediately after clamping (cases A1 and A2 for loads 1 and 2) or immediately after obtaining the homeostatic configuration (cases A0 and R0), a myograph test is simulated, as in the mouse experiments explained in section 2.1. For further reference, an overview of these six cases is given in Table 1.

**Table 1:** Clarification of the codes of the six cases for which a myograph experiment is modeled: A0, R0, A1, R1, A2, and R2.

	**Immediately after clamping**	**After 1 month healing**
Clamp load of 0.0 N	A0	R0
Clamp load of 0.6 N	A1	R1
Clamp load of 1.27 N	A2	R2

During the simulation, first, the axial boundary condition is released, as well as the intraluminal pressure to simulated excision, as shown by step 4 in Figure 4. Step 5 depicts the simulation of a myograph experiment as explained in section 2.1 similarly to Famaey et al. (2013). An undeformable rod with a radius of 0.15 mm in Abaqus is pushed into the arterty until the approximate required preload of 0.0133 N per mm length of the arterial segment is reached, assuming that the preload was set at 0.02 N for a length of approximately 1.5 mm in the actual experiments carried out by Geenens et al. (2016a). The rod is then fixed at this position, while 10^−6^ M PE, 10^−5^ M ACh, or 10^−6^ M NO is virtually added through field variables. A contact definition is prescribed between the rod and the inner surface of the artery. Pre-constriction due to PE and relaxation due to ACh or NO can then be observed according to the smooth muscle contraction model described in section 2.5.

#### 2.6.5. Remodeling Beyond 31 Days

In order to further examine the behavior of the remodeling model, the finite element analysis of section 2.6.3 was extended to a remodeling period of 91 days. The effect of slight adaptations to the model was investigated as well. The first adaptation is the assumption that synthetic SMCs do not redifferentiate into their contractile phenotype, such that Equations (17) and (19) only hold when Δλ is greater than or equal to zero. In the opposite case, only the inflammation level influences the SMC densities:

(36) $$\begin{array}{l} \frac{d\rho^{csmc}}{dt}=0\\ \frac{d\rho^{ssmc}}{dt}=K_{ic}^{smc}\phi^{ic}\rho^{ssmc}. \end{array}$$

The second adaptation is the assumption that collagen production is solely dependent on the amount of synthetic SMCs, while their production rate is not directly affected by the mechanical environment. Equation (15) then simplifies to

(37) $$\begin{array}{l} \Gamma \left(\tau \right)=\frac{\rho^{ssmc}\left(\tau \right)}{\rho_{0}^{ssmc}}. \end{array}$$

### 2.7. Model Parameters

An overview of all used parameter values is given in Tables 2, 3. A code number from 1 to 5 is attributed to every parameter, explaining the way its value was determined. We either used an exact value from the specified reference (1), or used a representative value from the reference, when for example a range was given based on the results of tests on multiple samples (2). Some parameter values are estimated (5), or the parameter is manually fitted to the experimental myograph data (see section 2.1), either with an idea of the order of magnitude from literature (4), or without (3).

**Table 2:** Overview of parameter values for constituent densities, passive and active material properties, pre-stretch values, damage parameters and remodeling parameters.

**Parameter**	**Value**	**References**
**INITIAL DENSITIES**		
$\rho_{0}^{elas}$	0.35	Bersi et al., 2016 (2)
$\rho_{0}^{coll}$	0.30	Bersi et al., 2016 (2)
$\rho_{0}^{csmc}$	0.30	Bersi et al., 2016 (2)
$\rho_{0}^{ssmc}$	0.05	Bersi et al., 2016 (2)
**PASSIVE MATERIAL PARAMETERS**		
*C*_10_	0.04 MPa	Bersi et al., 2016 (2)
*k*_1_	1.0 MPa	Bersi et al., 2016 (2)
*k*_2_	1.5	Bersi et al., 2016 (2)
κ	0.1	(5)
α	π/8 rad	(5)
**SMC PARAMETERS**		
μ^*csmc*^	0.42 MPa	Murtada et al., 2010 (4)
κ_*c*_	1.55 MPa	Murtada et al., 2010 (4)
**PRESTRETCHES**		
*g*_*ax*_	1.67	Bersi et al., 2016 (1)
*g*_*c*_	1.1	Bellini et al., 2014 (2)
**DAMAGE PARAMETERS**		
*m*^*csmc*^	1.0	(3)
*m*^*coll*^	20.0	(3)
*m*^*ec*^	0.38	(3)
**REMODELING PARAMETERS**		
$K_{qh}^{coll}$	log(2.0)/100 day^−1^	(3)
$K_{m}^{coll}$	26.64	(3)
$K_{pl}^{smc}$	4.0 day^−1^	(3)
*K*^*ec*^	0.08 day^−1^	(3)
$K_{dd}^{smc}$	1.6 day^−1^	(3)
$K_{ic}^{smc}$	0.01 day^−1^	(3)

(1) The exact value from the reference is used.

(2) A representative value from the reference is used.

(3) The parameter is manually fitted.

(4) The parameter is manually fitted in the same order of magnitude as the reference.

(5) *The parameter is estimated*.

**Table 3:** Overview of parameter values for the SMC contractility model.

**Parameter**	**Value**	**References**
**SMC CONTRACTILITY PARAMETERS**		
*k*_3_	0.4 s^−1^	Hai and Murphy, 1988 (1)
*k*_4_	0.1 s^−1^	Hai and Murphy, 1988 (1)
*k*_7_	0.01 s^−1^	Hai and Murphy, 1988 (1)
η	60.0 MPa·s	Murtada et al., 2010 (1)
*K*_*NO*_	8.0e-8 M	(3)
*K*_*PE*_	2.0e-7 M	(3)
${\left[Ca^{2+}\right]}_{hom}$	2.7e-7 M	Utz et al., 1999 (2)
α_*NO*_	1.4e-7 M	(3)
α_*PE*_	1.28e-7 M	(3)
α_*Ca*_	0.24	(3)
*K*_*CaCaM*_	1.78e-7 M	Murtada et al., 2010 (1)
*k*_2, *hom*_	0.5 s^−1^	Hai and Murphy, 1988 (1)
α_2_	0.1 s^−1^	(3)

(1) The exact value from the reference is used.

(2) A representative value from the reference is used.

(3) *The parameter is manually fitted*.

## 3. Results

### 3.1. Damage Due to Clamping

**Figure 5:**
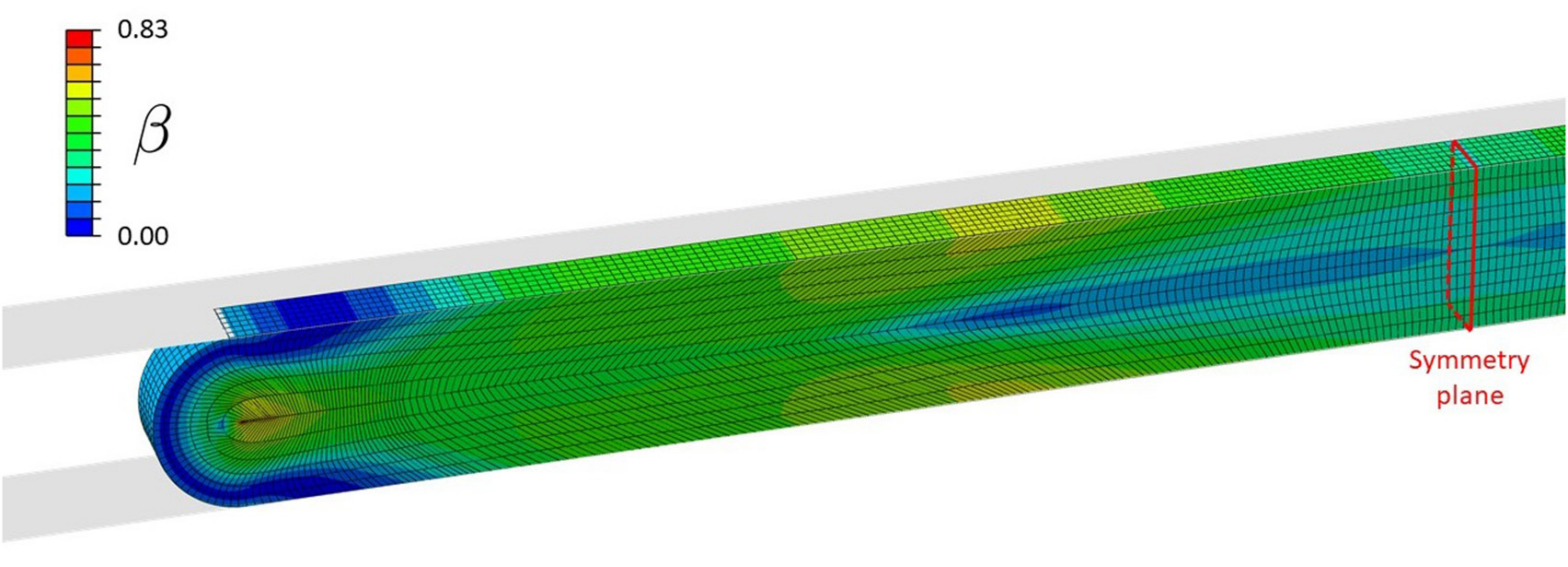
Distribution of β during clamping at a load level of 1.27 N, causing contractile SMC and endothelial damage.

Figure 5 shows the distribution of β (see Equation 10), defining the local loss of contractile SMC due to clamping. The highest damage is concentrated at the inner side of the wall at the edge of the clamp.

Table 4 gives an overview of the relative collagen, contractile SMC, and endothelium content acutely after clamping at the three different clamp loads (cases A0, A1, and A2). From this table it can be concluded that the difference between clamping at 0.6 and 1.27 N is small in terms of acute damage. There is approximately 70% loss of endothelium, 9% collagen loss, and 28% contractile SMC loss in both cases. The small difference in damage is due to a minimal required clamp displacement to increase the reaction force from 0.6 to 1.27 N, yielding only small stretch differences.

**Table 4:** The fraction of overall elastin, collagen, contractile SMC (cSMC), synthetic SMC (sSMC), and endothelium content with respect to their normal content.

**Case**	**A0**	**R0**	**A1**	**R1**	**A2**	**R2**
Elastin	1.0000	1.0000	1.0000	1.0000	1.0000	1.0000
Collagen	1.0000	1.0000	0.9076	1.6123	0.9071	1.6157
cSMC	1.0000	1.0000	0.7170	0.6539	0.7190	0.6575
sSMC	1.0000	1.0000	1.0000	1.7225	1.0000	1.7253
Endothelium	1.0000	1.0000	0.3113	0.8463	0.2719	0.8184
Inflammation	0.0000	0.0000	0.6887	0.1537	0.7281	0.1816

*Note that the level of inflammation is zero in the normal artery wall state, and has a maximal value of 1. Cases A0, A1, and A2 refer to the acute situation after clamping at three load levels (0.0, 0.6, and 1.27 N). R0, R1, and R2 correspond to the respective cases after 31 healing days*.

### 3.2. Remodeling

Table 4 also shows the situation after the simulated *in vivo* healing period of 31 days (cases R0, R1, and R2) using the presented remodeling model, taking into account cell differentiation, ECM production by synthetic cells and inflammation after clamping injury.

**Figure 6:**
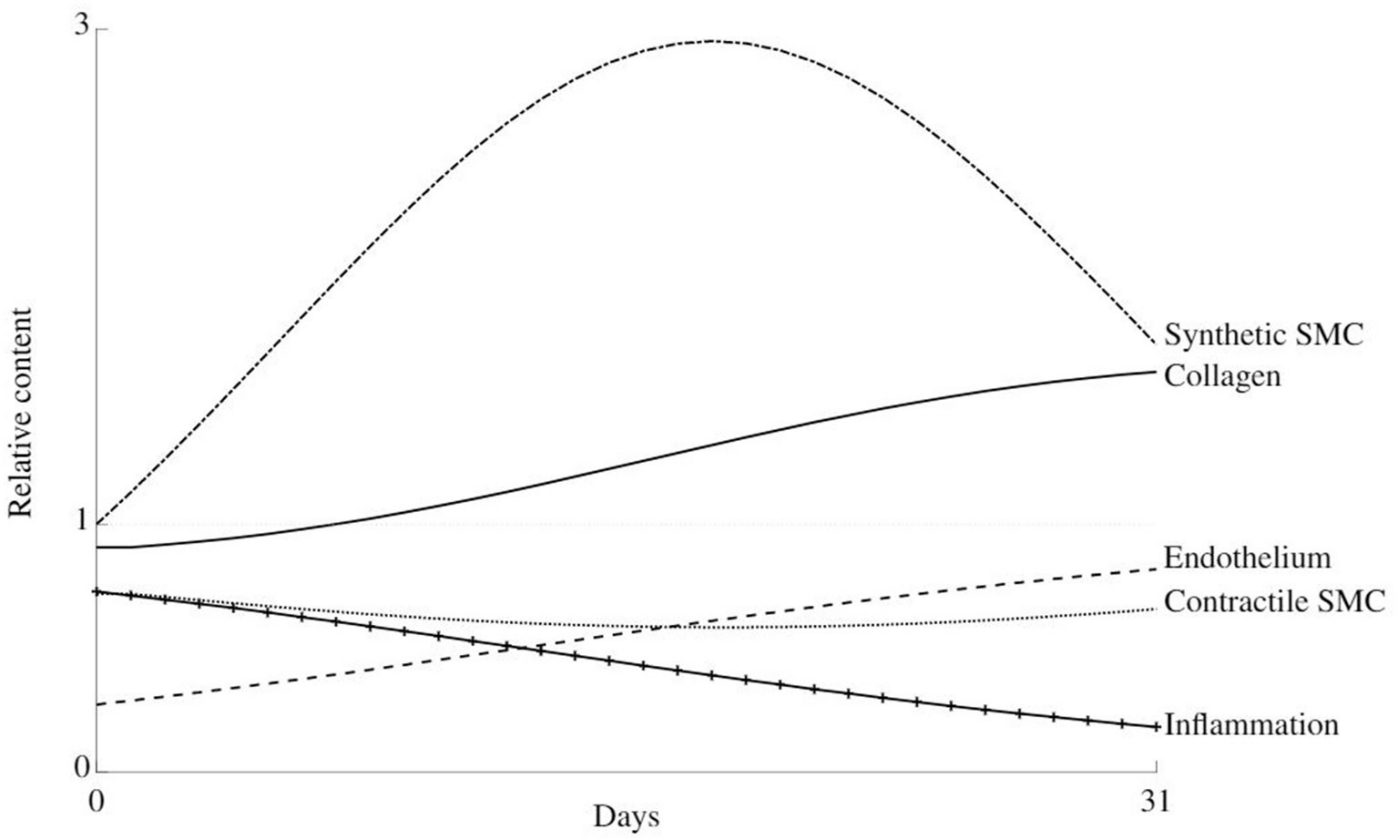
Thirty-one days evolution of the relative content of all considered constituents in the arterial wall during healing after damage due to clamping at load level 2.

Figure 6 shows the evolution of the total content of each constituent relative to its normal amount over a remodeling time of 31 days after damage due to clamping at 1.27 N. In other words, it shows the evolution from case A2 to case R2. Due to the similar level of damage at cases A1 and A2, as is clear from Table 4, the evolution from cases A1 to R1 resembles the one depicted in this figure.

There is an initial dedifferentiation of the cells from their contractile to synthetic phenotype due to an initial overstretching of the wall. Along, with the high initial inflammation level, this also causes the synthetic cells to proliferate, such that the collagen content increases. At about 14 days, the initial stiffness loss is compensated, and the collagen, synthetic, and contractile cell contents slowly return to their normal levels.

### 3.3. Myograph Test

**Figure 7:**
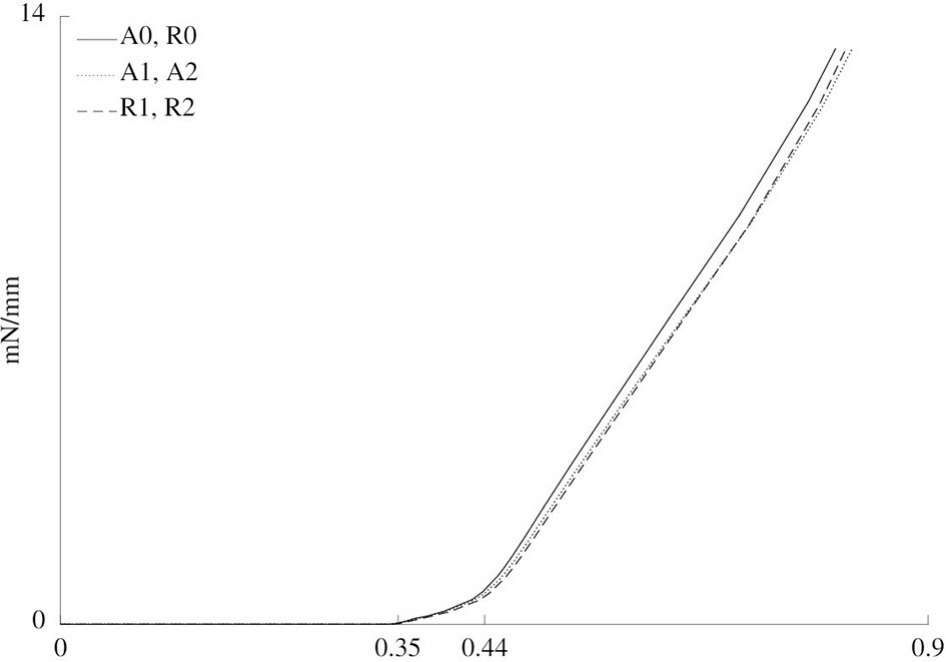
Normalized force vs. rod displacement for the six cases. Three different zones are discerned. From 0 to approximately 0.35 mm of displacement, the rod does not touch the sample yet, such that the force is zero. From approximately 0.35 to 0.44 mm, the cylindrical shape of the sample is straightened out. Finally, after approximately 0.44 mm, the sample is further stretched.

Figure 7 shows the normalized reaction force in the simulated rod while it moves toward the pre-load position before the addition of vasoactive substances. It indicates the overall stiffness of each material for each case. There was no discernible difference between cases A1 and A2 on the one hand and cases R1 and R2 on the other hand, as can be observed in Figure 7. Furthermore, the simulations do not show differences between cases A0 and R0.

**Figure 8:**
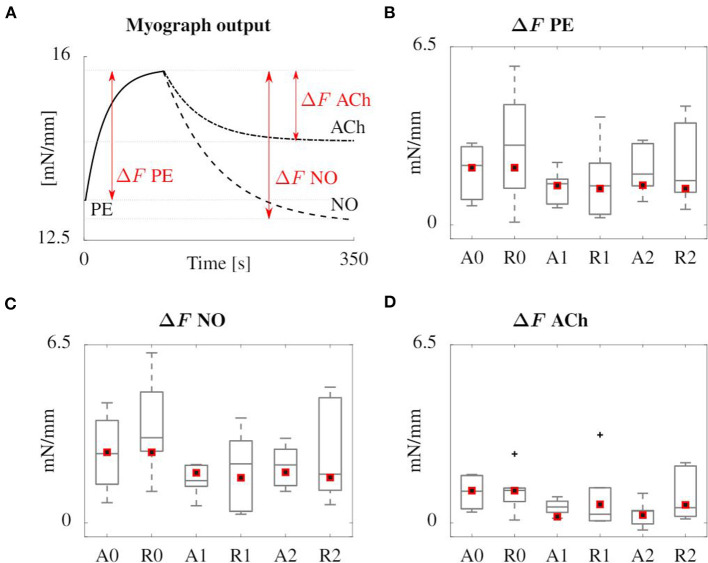
Overview of the results of the simulated (red squares) and experimental (boxplot) myograph results. **(A)** Representative scheme of the isometric force measured in the simulated myograph upon addition of vasoactive substances. **(B)** Force increase due to PE addition. **(C,D)** Subsequent force decrease after addition of NO and ACh, respectively. All forces are normalized with the axial length of the sample. The boxplots show the median values, the 25th and 75th percentiles, the total extent of the measurements without outliers (whiskers) and the outliers (crosses).

Figure 8 gives an overview of the results of the simulated myograph experiments upon the addition of vasoactive substances, compared to the results obtained on mouse arteries, as explained in section 2.1. The figure shows how the isometric force changes upon addition of PE, NO and ACh. PE drives an increased phosphorylation rate *k*_1_ = *k*_6_ of the myofilaments through an increased intracellular calcium level, inducing a vasoconstrictive effect. NO has the reverse effect on calcium and it also increases the dephosphorylation rate *k*_2_ = *k*_5_. ACh does not act directly on the contractile SMC, but triggers the endothelium to produce NO. Therefore, the vasodilating effect of ACh is smaller than that of NO.

### 3.4. Remodeling Beyond 31 Days

**Figure 9:**
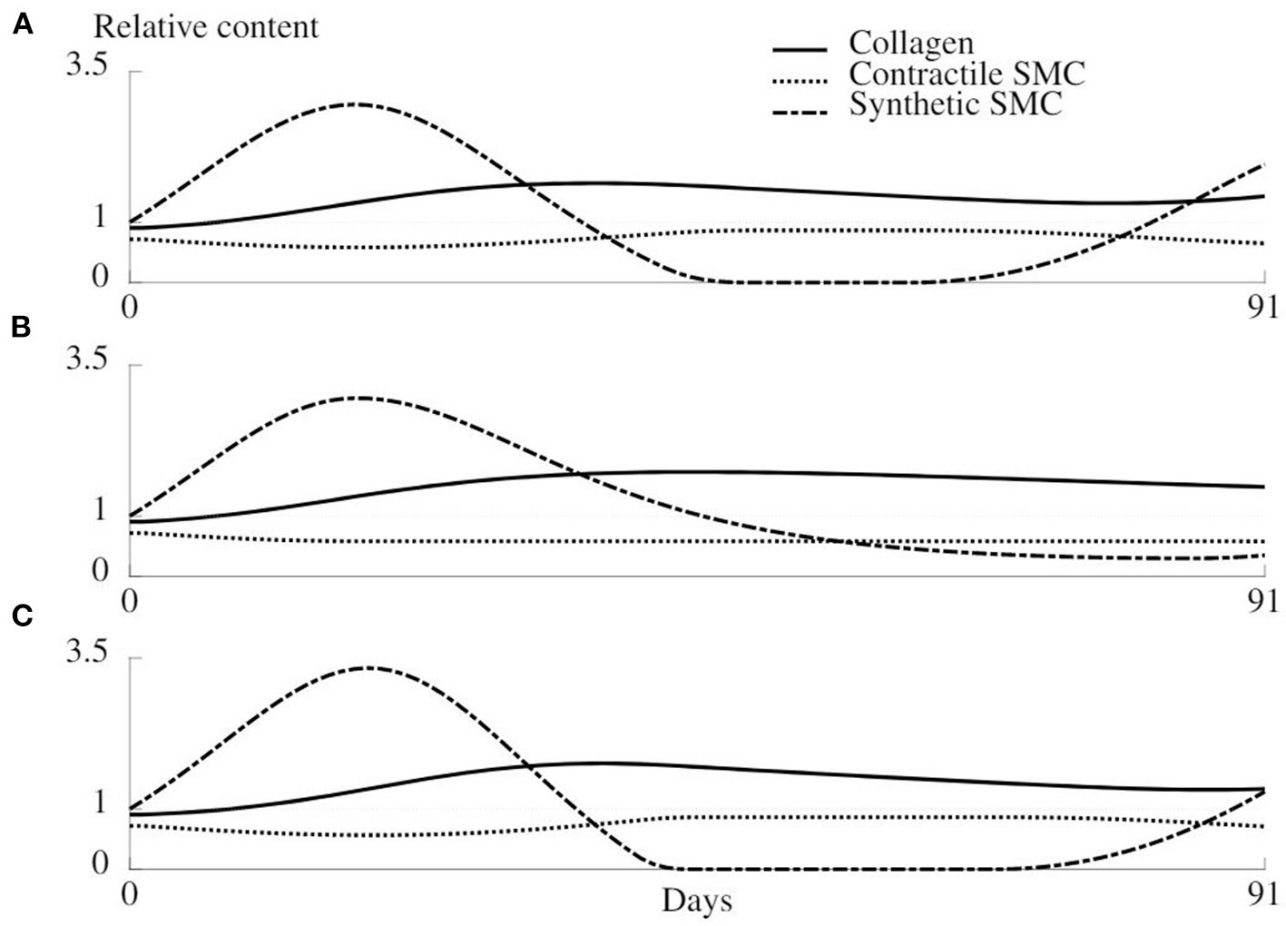
Evolution of the relative density of collagen, synthetic SMC, and contractile SMC during 91 days of healing for the original remodeling model **(A)** and two adapted versions **(B,C)**. **(A)** Original model as described in section 2.4. **(B)** The mechanical trigger that regulates the number of synthetic cells can only act by increasing the number of cells, and is otherwise deactivated. **(C)** The production of collagen is only related to the number of synthetic cells, not to the mechanical environment.

The evolution of relative collagen, synthetic cells, and contractile cells density over a remodeling period of 91 days is shown in Figure 9, for the original remodeling model (A) and two adapted models (B and C) as explained in section 2.6.5. Beyond 1 month, unnatural periodic behavior emerges when using the original model, caused by the initial extra loss of contractile SMC upon overstretching, causing an extra stiffness loss, and a delay in the increased collagen production through the proliferation of synthetic cells.

When synthetic cells do not redifferentiate into the contractile phenotype, a more rapid stabilization of the remodeling is observed (Figure 9B). However, the loss of contractile cells due to clamping overload will never be compensated in this case.

The results of the last variant of the remodeling model are shown in Figure 9C and show an increased oscillation of the synthetic cell density. Collagen production is not dependent on a mechanical stimulus anymore, such that a bigger increase in synthetic cell count is required to restore the collagen density, and along with it, restore the homeostatic mechanical environment.

## 4. Discussion

The aim of this study is to introduce a computational model predicting healing in arterial tissues subjected to mechanical overloading and damage, for instance after clamping. Three models are introduced for the *in silico* simulation of the experiments carried out by Geenens et al. (2016a): a damage model for clamping, a remodeling model to predict healing and a contractility model to simulate myograph experiments.

The contractility model is original as it is the first to take the vasoactive substances PE, NO, and ACh into account. Their respective influence on the rate of phosphorylation and dephosphorylation of myosin light chain leads to a reliable response in the simulation of a myograph experiment, as shown in Figure 8. The model is based on signaling pathways on the cellular level, dependent as well as independent on intracellullar calcium, as shown in Figure 3. The approach is different from the recent model presented by Murtada et al. (2016), in which the smooth muscle tone prediction was based on a structurally motivated model of the contractile unit. In their implementation, the response to an external factor, such as a change in loading or in the concentration of a vasoactive agent, is modeled as an evolving scaling factor for the myosin filament length. Before us, the continuum mechanics-based model of Murtada et al. (2017) was the only one that accounted for the dependency of the phophorylation rates on the diffusion of the vasoconstrictor potassium chloride (KCl) from the adventitia, although diffusion itself is neglected in the present study.

The remodeling model includes novel aspects of cell differentiation upon mechanical stimulus and the production of extracellular matrix by synthetic SMCs. This production is also dependent on a certain level of tissue inflammation, as for example done by Latorre and Humphrey (2018b). In the present approach however, the inflammation level is directly related to the damage and healing of the endothelium. Inflammation increases the synthetic cell proliferation, thus indirectly enhancing collagen production (Davis et al., 2003), as summarized in Figure 2. Hence, our remodeling model includes all the relevant biological processes and pathways, in contrast to more phenomenological models, where collagen turnover is directly related to a mechanical stimulus, such as in Baek et al. (2006); Alberto Figueroa et al. (2009); Valentín et al. (2013); Cyron et al. (2016); Braeu et al. (2017); Famaey et al. (2018), and Mousavi et al. (2019).

This more detailed description of SMC behavior in vascular healing and remodeling comes at an increased computational cost. Moreover, Figure 9 shows stability issues of the model in the form of unnatural temporal oscillations of the densities at longer time scales. A solution could be to neglect the transient effects and only consider the steady state, such as done by Latorre and Humphrey (2018a). Alternatively, we can include damping in the model to obtain a critical or overdamped dynamic system in order to avoid unnatural periodic behavior. From a mathematical point of view, the main limitation is the high number of parameters, as summarized in Tables 2, 3. Some parameters are determined based on previous works or based on their physical meaning, others were set in order to match experimental findings, mainly based on the tissue properties at 0 and 31 days of healing, which were however not sufficient to uniquely determine the parameter values.

Unfortunately, the currently available experimental data is not sufficient to proof its pilot application. It is likely that other parameter combinations would amount to the same results as shown in Figure 8. Nevertheless, the phenomenological nature of this new model is strongly reduced as compared to state-of-the-art models. A high number of parameters can be qualified as physics-based, such that their values can be obtained through the design of dedicated biochemical experimental set-ups. This will allow these parameter values to be measured with more certainty, or with smaller confidence intervals, capturing the individual differences and differences between tissue types, allowing a better focus of the parameter fitting process.

Constrained mixture models are generally computationally expensive due to their high memory use, inversely related to the length of the time step. To ensure the feasibility, we chose to use a time step of 1 day, where a time convergence study showed errors of <5% with respect to the situation with a time step of half a day. Also in an attempt to limit the computational cost, only a very short segment of artery is modeled and the defined boundary conditions cause a plane strain situation. Considering a longer segment, possibly along with a more realistic patient-specific geometry, would improve the reliability of the model, mainly near the edges of the clamp and near the edges of the excised sample during the myograph simulation. Using this very short artery segment allows to use a non-localized variable ϕ^*ec*^ that represents the overall intactness of the endothelium in the segment. Localizing the endothelial damage would greatly affect the complexity of the model, since diffusion of inflammatory agents and NO would need to be integrated. Similarly, taking into account the migration of SMC as an important mechanism in vascular remodeling, would increase the complexity as the remodeling in a certain integration point would be affected, not only by all variables defined in that specific location, but also by its surroundings. In a similar way, one could also consider re-endothelialization as a non-localized process of proliferation and migration of nearby endothelial cells. Including all these processes would increase the biofidelity of the model, although it is unclear to what extent, given the already many unknowns in the present version.

Furthermore, to further improve the remodeling model, an improved understanding of biological and biochemical phenomena is required. To this day, some unknowns, uncertainties and controversies remain. For example, it is unclear to what kind of mechanical stimulus cells react. There are indications that SMCs and fibroblasts have a preferred structural stiffness of the extracellular matrix and react based on deviations from this ideal value (Humphrey, 2008). On the other hand, certain signaling pathways are thought to be triggered by so-called baroreceptors, sensitive to mechanical stretch (Lacolley et al., 2017). Multiple studies have investigated the effects of cyclic straining of arterial tissue, as reviewed by Mantella et al. (2015). Some apparently contradictory results emerge. For example, Chang et al. (2003) observed an increased SMC proliferation under *in vitro* cyclic strain, while Morrow et al. (2005) and Guha et al. (2011) observed a decreased proliferation, potentially due to a different experimental design that mimics *in vivo* loading conditions better (Mantella et al., 2015). The widely accepted theory that precursor cells differentiate into synthetic cells and subsequently become fully differentiated contractile cells has been challenged recently as well, given that both phenotypes can be present in healthy tissues while maintaining vascular tone and tissue architecture (Rensen et al., 2007).

In summary, the presented models provide a detailed description of vascular SMC behavior under conditions of damage as well as at different concentrations of vasoactive agents. This allows us to study tissue healing and the effects of, for example, vasoactive or anti-proliferative drugs. However, there are still many unknowns regarding these phenomena, which is why more detailed and carefully designed experiments are needed in order to fully capture SMC behavior in all its aspects.

To conclude, in this study, a damage model, as well as a remodeling and cell contractility model were introduced, taking into account endothelial damage and healing, tissue inflammation, mechanosensing, extracellular matrix production and phenotype switching of SMCs. Using these models, *in vivo* clamping tests on mice aortas and subsequent healing and myograph tests, were simulated through finite element modeling. The results of the simulated myograph tests showed great resemblance to the results of the actual experiments. This detailed mechanobiological description of vascular SMC behavior can be clinically relevant to enable *in silico* investigations of drug effects. However, the results show that there is still a need for an improved biological and biochemical fundamental understanding to reliably capture vascular SMC mechanobiology at all the relevant spatio-temporal scales.

## Data Availability Statement

The raw data supporting the conclusions of this article will be made available by the authors, without undue reservation.

## Ethics Statement

The animal study was reviewed and approved by Ethical committee for animal experimentation (ECD), KU Leuven, Belgium.

## Author Contributions

LM, SA, and NF conceived the presented ideas. LM developed and implemented the presented algorithms and wrote the manuscript. JV, SA, and NF reviewed the manuscript and reviewed the computational methods. SA and NF supervised the project. All authors contributed to the article and approved the submitted version.

## Conflict of Interest

The authors declare that the research was conducted in the absence of any commercial or financial relationships that could be construed as a potential conflict of interest.
